# Repeatability of brown adipose tissue measurements on FDG PET/CT following a simple cooling procedure for BAT activation

**DOI:** 10.1371/journal.pone.0214765

**Published:** 2019-04-17

**Authors:** John P. Crandall, Prateek Gajwani, Joo H. O., Daniel D. Mawhinney, Fred Sterzer, Richard L. Wahl

**Affiliations:** 1 Mallinckrodt Institute of Radiology, Washington University in St. Louis, St. Louis, Missouri, United States of America; 2 Dana Center for Preventive Ophthalmology, The Wilmer Eye Institute, Johns Hopkins University School of Medicine, Baltimore, Maryland, United States of America; 3 Department of Radiology, College of Medicine, Seoul St. Mary's Hospital, The Catholic University of Korea, Seoul, Korea; 4 MMTC, Inc., Princeton, New Jersey, United States of America; 5 The Russell H. Morgan Department of Radiology and Radiological Science, Johns Hopkins Medical Institutions, Baltimore, Maryland, United States of America; St. Joseph's Hospital and Medical Center, UNITED STATES

## Abstract

Brown Adipose Tissue (BAT) is present in a significant number of adult humans and can be activated by exposure to cold. Measurement of active BAT presence, activity, and volume are desirable for determining the efficacy of potential treatments intended to activate BAT. The repeatability of 18F-fluorodeoxyglucose (FDG) positron emission tomography (PET) measurements of BAT presence, activity, and volume under controlled conditions has not been extensively studied. Eleven female volunteers underwent double baseline FDG PET imaging performed following a simple, regional cold intervention intended to activate brown fat. The cold intervention involved the lightly-clothed participants intermittently placing their feet on a block of ice while sitting in a cooled room. A repeat study was performed under the same conditions within a target of two weeks. FDG scans were obtained and maximum standardized uptake value adjusted for lean body mass (SULmax), CT Hounsfield units (HU), BAT metabolic volume (BMV), and total BAT glycolysis (TBG) were determined according to the Brown Adipose Reporting Criteria in Imaging STudies (BARCIST) 1.0. A Lin’s concordance correlation (CCC) of 0.80 was found for BMV between test and retest imaging. Intersession BAT SULmax was significantly correlated (r = 0.54; p < 0.05). The session #1 mean SULmax of 4.92 ± 4.49 g/mL was not significantly different from that of session #2 with a mean SULmax of 7.19 ± 7.34 g/mL (p = 0.16). BAT SULmax was highly correlated with BMV in test and retest studies (r ≥ 0.96, p < 0.001). Using a simplified ice-block cooling method, BAT was activated in the majority (9/11) of a group of young, lean female participants. Quantitative assessments of BAT SUL and BMV were not substantially different between test and retest imaging, but individual BMV could vary considerably. Intrasession BMV and SULmax were strongly correlated. The variability in estimates of BAT activity and volume on test-retest with FDG should inform sample size choice in studies quantifying BAT physiology and support the dynamic metabolic characteristics of this tissue. A more sophisticated cooling method potentially may reduce variations in test-retest BAT studies.

## Introduction

Obesity is a global epidemic with significant health consequences. According to the Centers for Disease Control and Prevention (CDC), the prevalence of adult Americans considered overweight and obese was 71.1% and 38.9%, respectively, between 2013 and 2016 [1]. One study projects that 85% of Americans could be obese or overweight by 2030 [2]. Obesity is a risk factor for numerous conditions including high blood pressure, type II diabetes, and some cancers [3]. Added to the health impact is the economic burden of obesity and obesity-related conditions, which has been estimated to consume 21% of healthcare expenditures in the United States [4]. Weight management has been shown to aide in the reversal of obesity-related conditions such as type II diabetes, but current weight-loss strategies have shown limited efficacy and alternative weight management tools may be necessary to help reduce the excessive burden of obesity [5].

The human body has two primary types of adipose tissue: white adipose tissue (WAT) and brown adipose tissue (BAT) [6]. Lipid storage for subsequent use during caloric shortage is the principal WAT function. In mammals, BAT plays a key role in the regulation of energy and metabolism and helps protect against hypothermia via increased thermogenesis [7]. BAT uncoupling protein-1 (UCP1), which dissociates oxidative phosphorylation from ATP production and releases the energy stored in the mitochondrial proton electrochemical gradient as heat. UCP-1 knock out mice are obese, consistent with a role of BAT in maintaining caloric balance. BAT activation of lipolysis and the associated thermogenesis has been shown to protect rodents against obesity and diabetes [8].

The degree to which BAT is present and active in adult humans was unclear until the somewhat recent introduction of imaging modalities such as [18F]fluorodeoxyglucose (FDG) positron emission tomography combined with computed tomography (PET/CT) and selective tissue sampling, which have confirmed that functional human BAT exists into adulthood [9–11]. Furthermore, the inverse correlation between active BAT and overall adiposity may imply that BAT plays a central role in human energy regulation [12]. Thus, various obesity interventions that aim to increase BAT volume and activity are currently being studied [13–17].

PET/CT using FDG has become the gold standard for the detection of active BAT in humans [18]. Increased uptake of FDG in BAT, quantified using the standardized uptake value (SUV) parameter, indicates greater glucose utilization and has been shown in correlative studies with 15O-oxygen and 11C-acetate, to be associated with increased oxidative metabolism and thermogenesis in BAT [19–21]. FDG uptake in malignancies has been shown to exhibit a high degree of reproducibility on PET/CT [22, 23]. Nahmias et al found a correlation coefficient of 0.99 for serial measurements of mean standardized uptake value (SUVmean) performed within five days [24]. In recurrent ovarian cancer patients, one study found FDG uptake measurements to have repeatability coefficients of 16.3% and 17.3% for SUVmean and SUVmax, respectively [25]. A combined analysis of two multicenter trials showed that, based on repeated measurements, decreases in FDG uptake by more than 30% and increases by more than 40% are likely to reflect true metabolic changes, in patients with non-small cell lung cancer [26]. As FDG PET/CT appears quite reliable in the oncology setting, it is reasonable to assume that variability in test-retest imaging of BAT is likely due to intrinsic changes in the BAT rather than variability associated with the imaging method.

The advent of functional metabolic imaging has fueled a resurgence of interest in human BAT. Development of pharmaceuticals and other methods to increase BAT volume and duration of activity are ongoing. However, in order to assess the efficacy of methods designed to increase BAT activation, one must have an idea of the repeatability of the FDG signal in brown adipose tissue. To date, a prospective, systematic assessment of the repeatability of the FDG signal in brown adipose tissue has not been carried out. The goal of this study (which was part of a larger study evaluating the operating characteristics of a microwave radiometer in the assessment of BAT; PMID: 29439011) was to evaluate the test-retest reliability of FDG PET/CT in measuring BAT glucose metabolism in subjects exposed to a simple regional, inexpensive cooling procedure, intermittent placement of the subjects feet on an ice-block, on different occasions.

## Materials and methods

This prospective study was approved by the Johns Hopkins Medicine Institutional Review Board (IRB approval NA_00050285) and conducted according to the principles expressed in the Declaration of Helsinki. Written, informed consent was obtained from all participants. Healthy volunteers between ages 18 to 35 with a body mass index (BMI) less than 25 kg/m^2^ were eligible for the study. Considering the age and BMI criteria for the study, those enrolled would not be considered representative of the general population. Volunteers were recruited using flyers placed in various places on the Johns Hopkins medical campus. Subjects received modest financial compensation for their investment of time. Pertinent exclusion criteria included known diabetes mellitus, the use of drugs known to modulate BAT activity (e.g. beta–blockers and certain psychotropic medications), a history of cold-related injury, and the use of nicotine-containing products. Pregnant or breast-feeding individuals were also excluded from the study.

All PET/CT images were acquired using the Discovery ST PET/CT system (GE Healthcare). Static PET images were generated using an ordered-subset expectation maximization reconstruction (OSEM). Participants were asked to avoid high carbohydrate meals and strenuous activity within 24 hours prior to both study visits. They were also instructed to fast for at least 6 hours prior to administration of FDG (PETNET Solutions INC., Knoxville, TN). All participants underwent a “BAT maximization” cooling protocol followed by intravenous injection of FDG (mean injected dose of 258.4 ± 36.4 MBq) was followed by a mean uptake period of 61.2 ± 5.5 minutes and then whole-body FDG PET/CT imaging. This process was then repeated within a target of 2 to 14 days following the first session. The BAT maximization protocol utilized in this study was based on the method described previously by Saito et al and consisted of cold stimulation in the form of a lightly clothed subject placed in a cooled room (18.1°C—20.0°C) as well as having the participants intermittently place their feet on a block of ice (*4*). Once cold stimulation began, participants were asked to place their feet on the block of ice for 4 minutes, followed by one minute of rest. The ice-block was covered with a disposable blue paper pad so the skin did not make direct contact with the ice. Participants wore a hospital gown (clo value of 0.44) and thin cotton hospital pants (clo value of 0.46). The cold stimulation went on for a target of 1 hour until the administration of FDG and continued during the FDG uptake period of about 1 hour (i.e. approximately 2 total hours of cold stimulation). A primary goal of this protocol was to cool the participant, but to minimize shivering, which could potentially confound the results of the radiometer and the PET/CT. To that end, volunteers were monitored throughout the session for signs of shivering. If shivering was detected by the researcher or reported by the volunteer, the cooling was modified by reducing the amount of time the participant’s feet were on the ice until shivering ceased. Cold stimulation was discontinued during PET/CT imaging. The patient’s body temperature (oral) was also monitored at regular intervals.

### PET/CT image analysis

A single, trained BAT biologist used a clinical imaging workstation (Mirada XD3, Mirada Medical, Denver, CO) for determination of metabolic values and to qualitatively analyze the PET/CT images. Supraclavicular areas expected to contain activated BAT were identified and SUV (adjusted for lean body mass—SUL), CT Hounsfield unit values, BAT metabolic volume (BMV), and total BAT glycolosis (TBG) determined. Per the BARCIST 1.0 criteria, a SULmax value of 1.2 g/mL or greater and Hounsfield unit range of -190 to -10, in regions expected to contain BAT, were used together as cutoffs to identify active BAT. When active BAT was not able to be identified, a “fat mask” was created in the supraclavicular region where BAT is normally found (and where uptake values are generally highest in BAT) and a SULmax was extracted using a previously described method [27]. A subset of participants were selected at random and analyzed by a second experienced BAT biologist/imaging specialist in order to estimate inter-rater reliability.

### Statistical considerations

Repeatability of the BAT signal was assessed using paired t-tests, Wilcoxon signed-rank tests, Lin’s concordance correlation coefficient (CCC), and reproducibility coefficients. A CCC value of 0.8 was considered repeatable and the coefficient was calculated using the following formula:

$$\frac{2\sigma_{x}\sigma_{y}}{\sigma_{x}^{2}+\sigma_{y}^{2}+{\left(\mu_{x}-\mu_{y}\right)}^{2}}$$

Where σ_x_ is the variance of session 1, σ_y_ is the variance of session 2, μ_x_ is the mean of session 1, and μ_y_ is the mean of session 2. Inter-rater reliability was assessed using an intraclass correlation coefficient (ICC), which was calculated using the following formula [28]:

$$\frac{\sigma_{x}^{2}}{\sigma_{x}^{2}+\sigma_{y}^{2}}$$

A within-subject coefficient of variation (wCV) was also calculated for each metric studied by dividing the standard deviation of the relative test-retest differences by the square root of two. Relative differences were calculated using the following formula:

$$\frac{S2-S1}{(S1+S2)/2}\times 100$$

Where S1 and S2 are study 1 values and study 2 values, respectively. In all analyses, a *P* value of less than 0.05 was considered statistically significant. Descriptive statistics were calculated using Microsoft Excel and further analyses were performed with Prism 4.0 (Graphpad Software).

## Results

**Fig 1:**
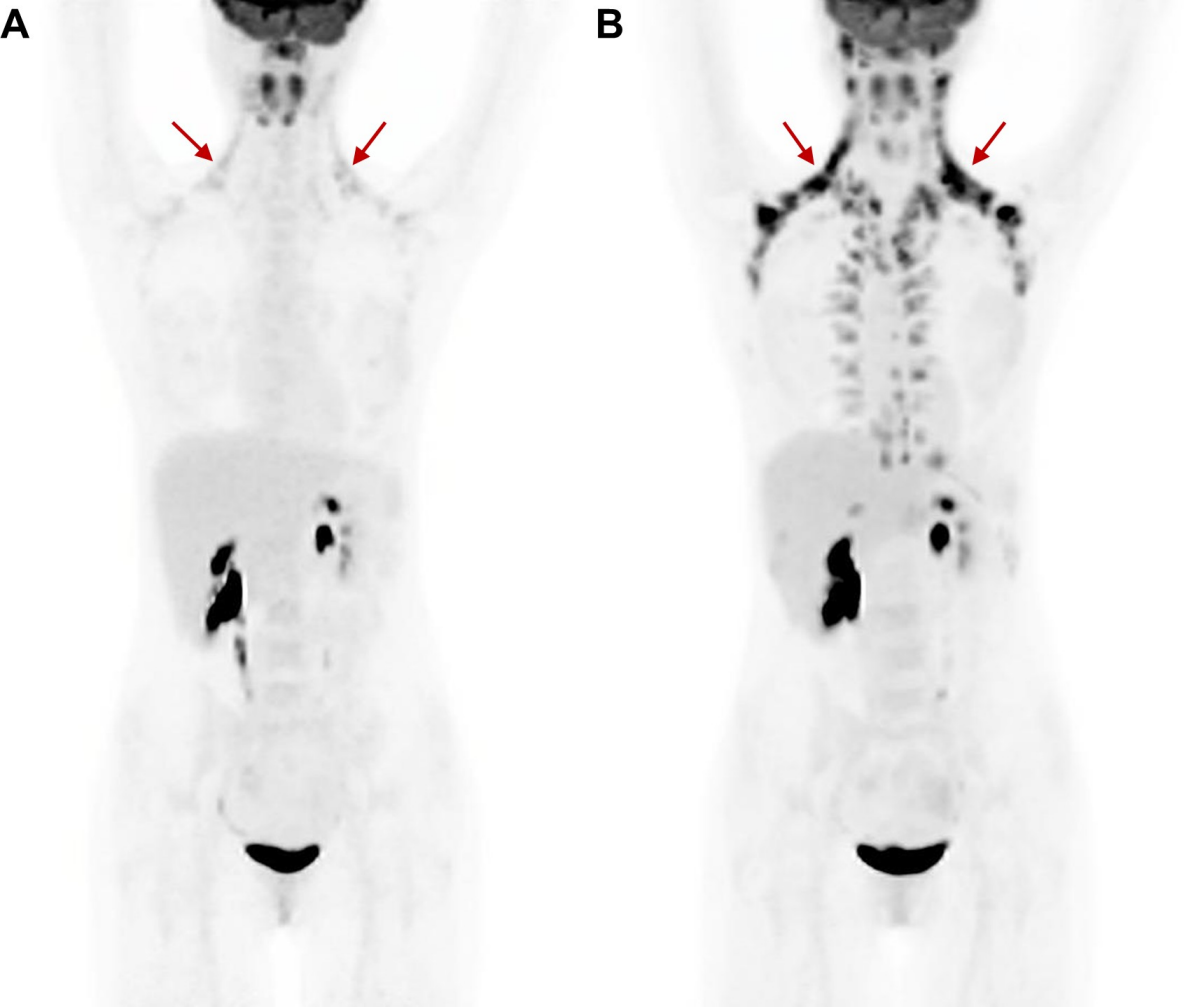
Representative PET/CT images. Active BAT is seen in both test (A) and retest (B) studies. Primary supraclavicular active BAT depots are indicated by red arrows.

Eleven participants were prospectively enrolled between March, 2012 and March, 2013 at the Johns Hopkins medical campus in Baltimore, MD. Participant characteristics are described in Table 1. All volunteers underwent two sessions that each included the BAT maximization procedure followed closely by FDG PET/CT imaging. Representative PET images displaying active BAT following both cooling sessions are shown in Fig 1. Two participants exhibited no BAT activity qualitatively on PET/CT during the baseline session and neither exhibited BAT activity during the repeat session. Additionally, two participants exhibited BAT activity during the baseline session but did not have active BAT during the repeat session. The median and range between BAT maximization sessions was 13 days and 2–34 days, respectively. All sessions were performed during months with cooler temperatures (i.e. November–May), with the exception of one performed in August, which did not result in activated brown fat during the BAT maximization sessions.

**Table 1:** Participant characteristics.

Participant	Age (yrs)	Race^a^	Ethnicity^b^	Body Weight (kg)	Height (m)	BMI (kg/m^2^)	Days Between Test and Retest Imaging
A	22	A	N	61	1.7	21.1	12
B	28	W	N	59	1.68	20.9	13
C	21	A	N	54	1.62	20.6	19
D	29	W	N	52	1.65	19.1	3
E	26	W	N	57	1.73	19	2
F	24	B	N	48	1.68	17	6
G	19	W	H	62	1.75	20.2	34
H	25	B	N	63	1.73	21	14
I	20	W	N	57	1.73	19	13
J	27	W	N	54	1.68	19.1	9
K	22	W	N	54	1.62	20.6	14
Median (IQR)^c^	24 (5.1)			57 (6.0)	1.68 (0.06)	20.2 (1.7)	13 (6.5)

^a^Asian, African American and Caucasian races are denoted by A, B, and W, respectively.

^b^Hispanic and non-Hispanic ethnicities are denoted by H and N, respectively.

^c^Median values and interquartile ranges (IQR) are reported for quantitative characteristics.

Shivering was observed or reported in 4/9 BAT-positive participants and was then minimized or eliminated in all 4 participants using the modified cooling protocol. When a participant reported or was observed shivering during the first cooling session, the cooling protocol was not modified during the second cooling session (i.e. all participants began with the same cooling protocol during both sessions).

**Fig 2:**
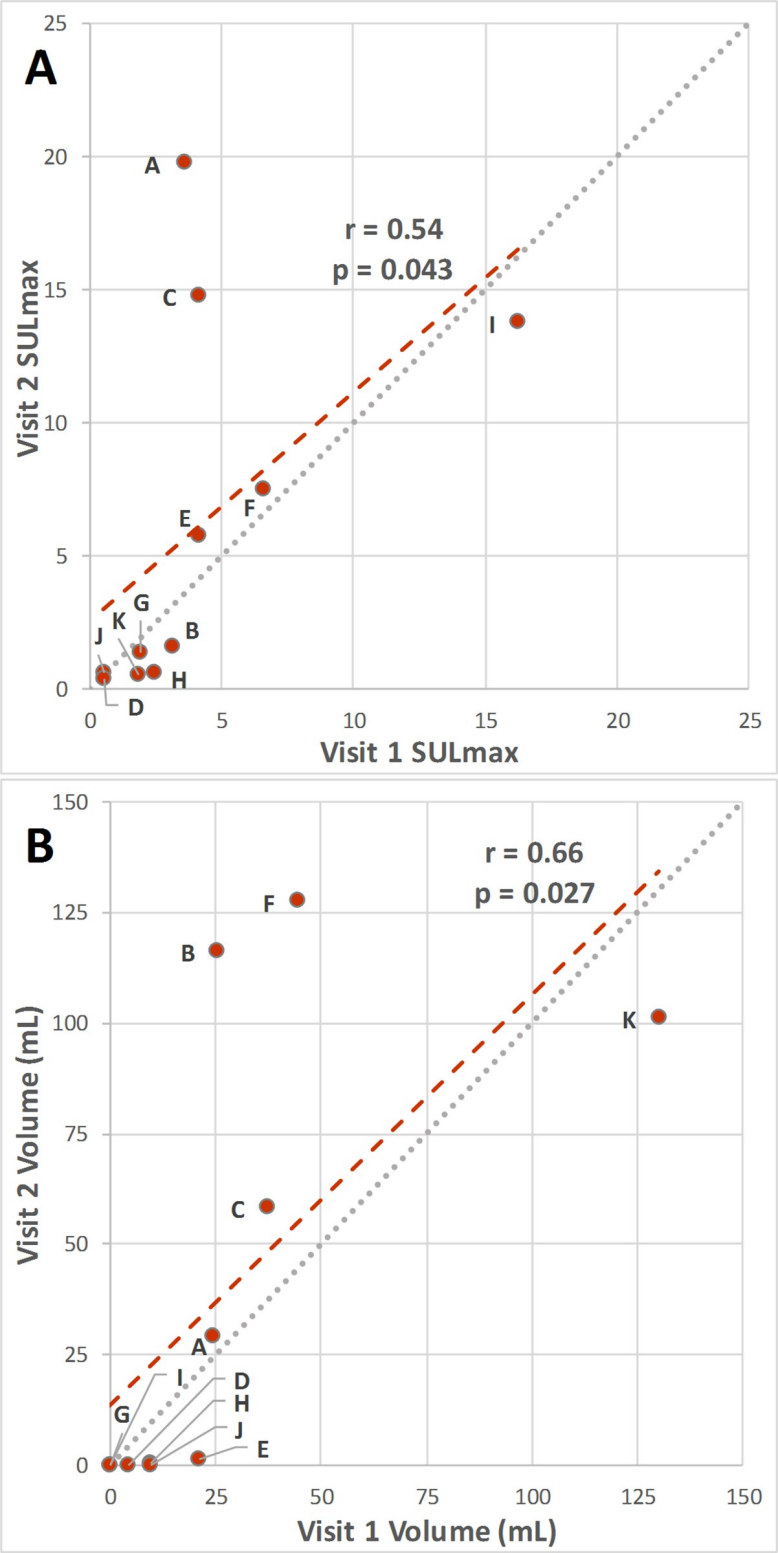
SULmax and BMV scatterplots showing the correlation between test and retest studies.

**Fig 3:**
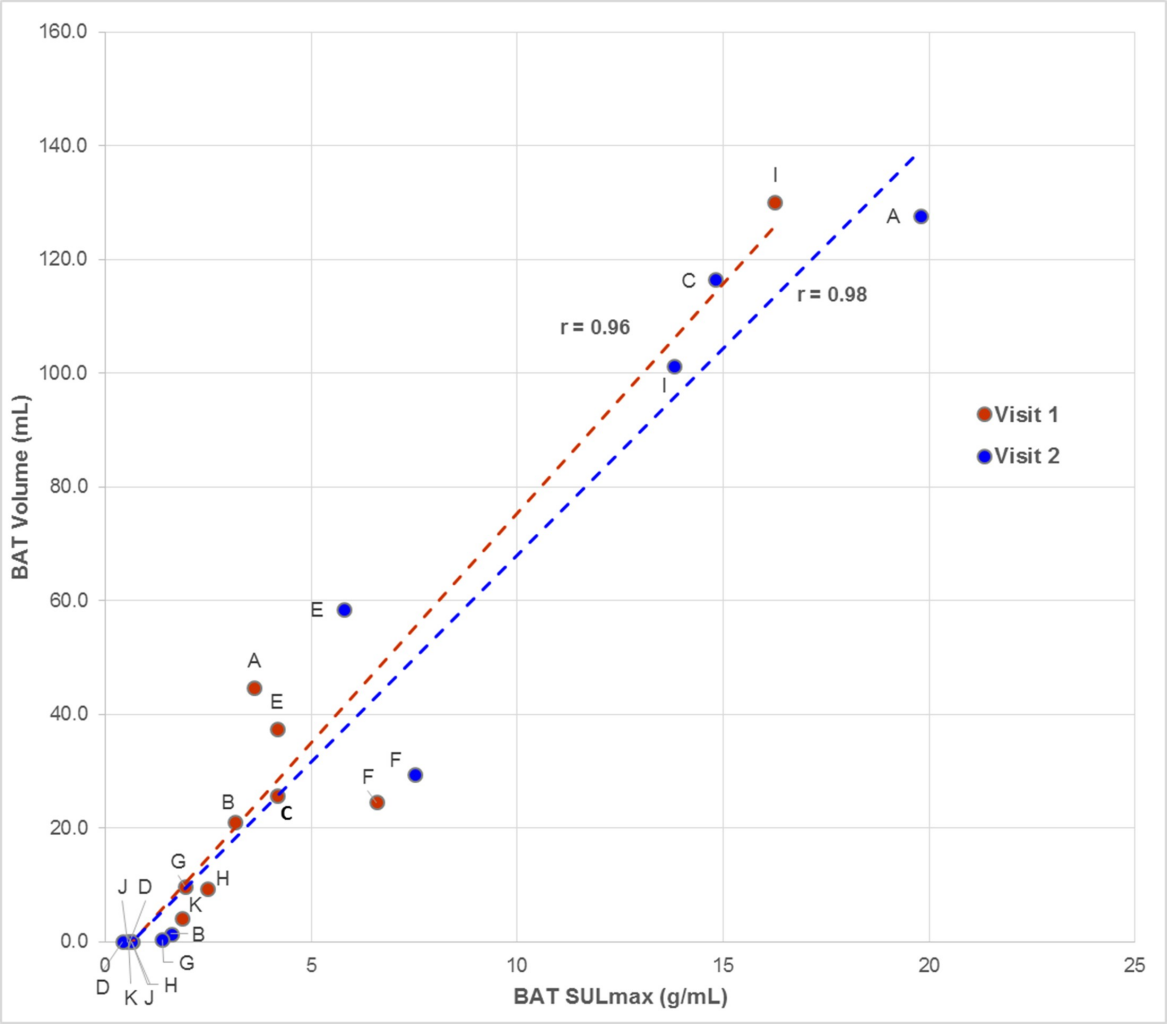
BAT volume and SULmax were highly correlated during each visit. A higher SULmax was strongly correlated with a higher BMV during test and retest visits. Data points are labelled using the identifiers in Table 1.

Average values of active BAT SULmax, BMV, HUmean, and TBG for both imaging sessions are listed in Table 2, along with the mean relative differences between test and retest studies and the wCV for each metric. Regression analysis showed BMV and SULmax were both significantly correlated between visit 1 and visit 2 (**Fig 2A and 2B**) with correlation coefficients of 0.66 and 0.54, respectively. BAT TLG was poorly correlated between the test and retest imaging sessions (r = 0.20; p = 0.56). BAT SULmax and BMV were also strongly correlated, with a higher BMV generally corresponding to a higher SULmax during both sessions (**Fig 3**). Using a CCC value of 0.80 as a cutoff, BMV was repeatable with a CCC of 0.80. SULmax, HUmean, and TBG were not considered to be as repeatable (CCC values of 0.69, 0.59, and 0.12, respectively). Inter-rater reliability was assessed using a subset of participants and ICC values of 1.00 and 0.96 were calculated for BAT SULmax and volume, respectively.

**Table 2:** Average BAT measurement values per visit and selected repeatability measures.

	Session #1	Session #2		
	*median (IQR)*	*median (IQR)*	μ_*MRD*_ *(SD)*	*wCV (%)*
**SULmax (g/mL)**	3.2 (2.3)	1.6 (10.0)	2.1 (73.7)	52.1
**Volume (mL)**	21.1 (24.7)	1.4 (79.9)	-45.5 (122.9)	86.9
**CT HUmean**	-54.5 (11.4)	-52.0 (10.4)	-25.7 (43.2)	30.5
**TLG**	32.0 (48.2)	1.8 (128.8)	-10.4 (147.0)	104.0

Regression analysis shows SULmax (A) and BMV (B) were each significantly correlated between test and retest studies. Data points are labelled using identifiers from Table 1.

### BAT SULmax repeatability

**Fig 4:**
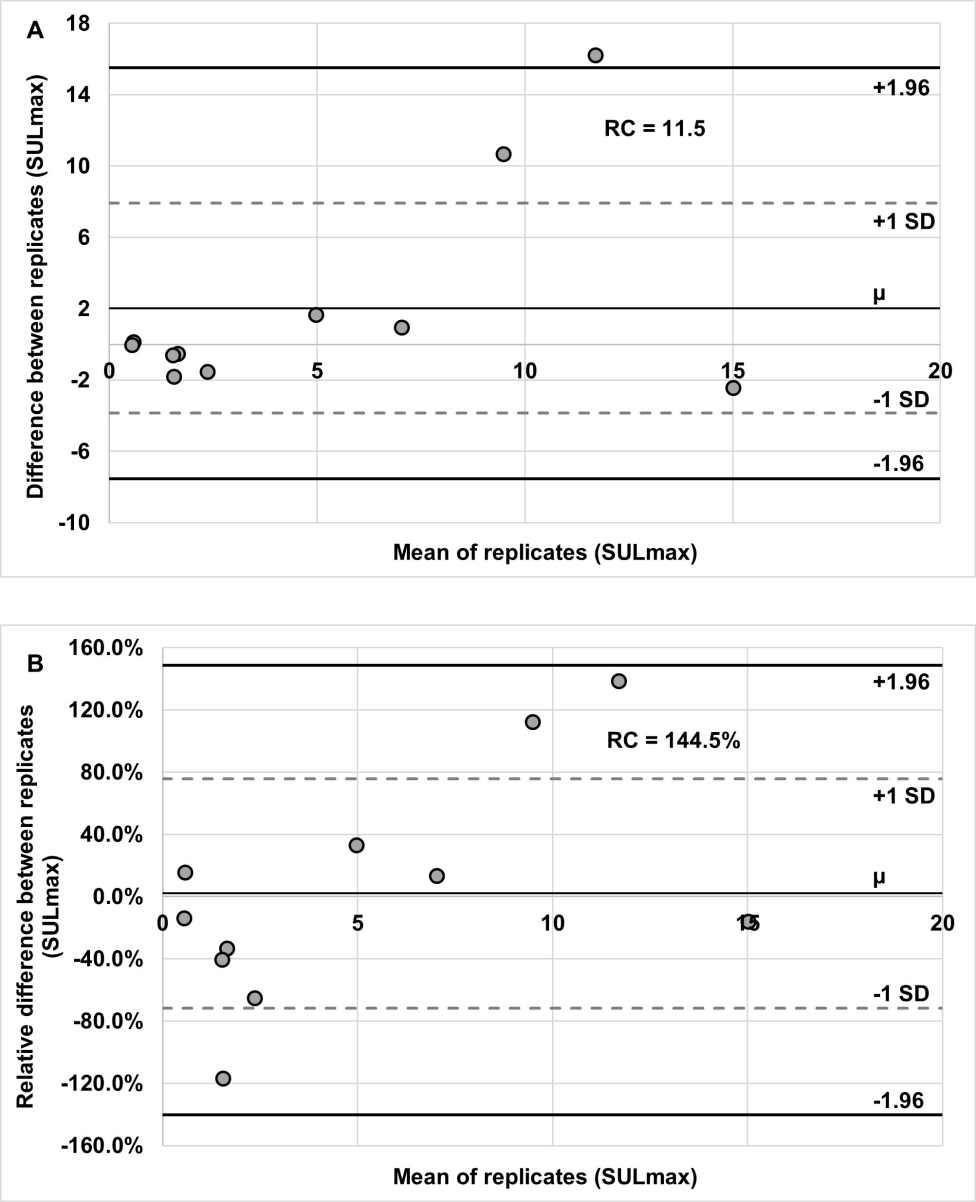
Bland-Altman plots showing the repeatability of BAT SULmax. Replicate means are plotted against both difference (A) and relative difference (B). Repeatability coefficient values are included in each chart.

Bland-Altman analysis revealed a repeatability coefficient of activated BAT SULmax of 11.5 g/mL (LOR: -7.6, 15.5), based on the difference between test and retest studies (**Fig 4A).** A repeatability coefficient for activated BAT SULmax, based on the relative different between test and retest visits, was144.5% (LOR: -140.4%, 148.6%) (**Fig 4B**). The intraclass correlation coefficient was 0.48 (95% CI: 0.39–0.57). A within-subject coefficient of variation of 52.1% was calculated for SULmax. The session #1 mean SULmax of 4.92 ± 4.49 g/mL was not significantly different from that of session #2 with a mean SULmax of 7.19 ± 7.34 g/mL (p = 0.16). A Wilcoxon signed-rank test indicated that test and retest SULmax did not differ significantly (p = 0.99).

### BMV repeatability

**Fig 5:**
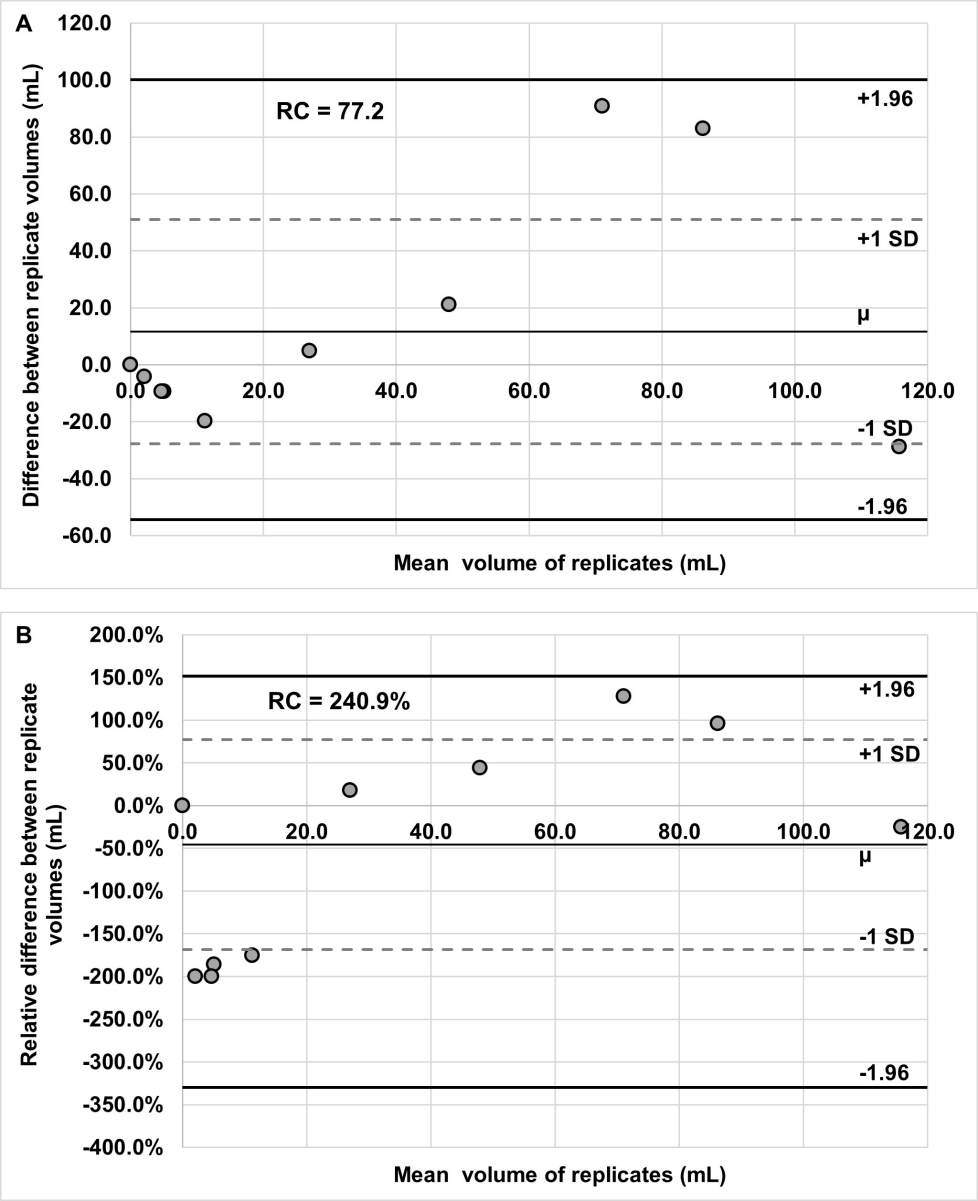
Bland-Altman plots showing BMV repeatability. Replicate means are plotted against both difference (A) and relative difference (B). Repeatability coefficient values are included in each chart.

Bland-Altman analysis revealed a repeatability coefficient for activated BMV of 77.2 (LOR: -54.4, 100.0), based on the difference between test and retest visits (**Fig 5A**). The repeatability coefficient for activated BMV, based on the relative difference between test and retest visits, was 240.9% (LOR: -330.0%, 151.7%; **Fig 5B**) and the wCV was 86.9%. The intraclass correlation coefficient was 0.62 (95% CI: 0.50–0.74). A Wilcoxon signed-rank test indicated no significant difference in BMV between test and retest visits (p = 0.82).

## Discussion

The most common technique used to image activated brown adipose tissue is currently FDG PET/CT. The repeatability of FDG uptake in activated BAT has not been extensively investigated. Various interventions intended to increase the volume and metabolic activity of BAT are currently being evaluated as obesity or diabetes treatments [29–31]. Given the dynamic nature of the brown adipose tissue, determining what change in metabolic status of BAT represents a true change in BAT activity or volume will help inform the design of studies assessing possible treatment strategies, in which changes in BAT volume or activity may be expected to precede other physiological markers of a successful anti-obesity therapy, such as reduction in BMI or blood pressure. As such, the test-retest reliability of FDG PET/CT in measuring active BAT is necessary information if one is to assess the effect of an intervention on brown fat activity by PET imaging. In the current study, BAT was activated, as assessed by FDG PET, on scans from at least one time point, in 9 of 11 healthy participants and was assessed by the BARCIST 1.0 criteria, by utilizing a simple cold-exposure technique that included readily available and inexpensive materials. The majority of volunteers (7/11) showed activated BAT on both test and retest scans by BARCIST. The qualitative assessments of the presence/absence of activated BAT activity were reasonably consistent, but other quantitative measurements of BAT activity showed somewhat or much lower repeatability when compared with reports from studies of FDG test-retest reliability in malignant tumors.

The SUV measurement extracted from PET images has become a primary metric for tumor quantification and SUV values in tumors have been shown to be quite repeatable [22, 25, 32]. One meta-analysis of FDG repeatability, including 163 lesions in 86 patients with various tumor types, found a SUVmax ICC of 0.90 [23]. A recent literature review found the mean SUVmax wCV from repeatability studies of FDG PET was 10.96 ± 3.32% [33]. Groshar et al enrolled 30 patients with varying cancer types and found a 95% RC of 29.4% for SUVmax, which is considerably lower than the 144.5% value we observed in BAT using our activation method [34]. In the context of obesity intervention assessment, this means an increase of SUVmax of more than 144.5% would be necessary in order to be reasonably sure there was truly more FDG uptake in BAT. This would be considered a very large change in tumor SUVmax, but it may not represent an unreasonable target for an increase in BAT activity, especially since the target population for an obesity intervention would have high BMIs, which has been shown to be inversely correlated with active BAT [35]. Therefore, in many cases, a successful BAT therapy may cause a change in obese patients from near zero to much higher BAT SUV values. Future studies on the repeatability of BAT in higher BMI populations would be of value, as lower baseline levels of BAT may result in greater test-retest variability. The large CV for BAT suggests that in an individual patient, it would be difficult to confirm a true decrease in BAT activity with an intervention, though it would be possible in populations.

BMV was the only quantitative metric found to have a CCC value that could be considered repeatable by using the .8 cut off for CCC repeatability (0.80). However, the 95% RC of 240.9% was still substantially higher than has been found in studies of metabolic tumor volume (MTV) repeatability in cancer. Kramer et al found a 30.9% RC for the volumes of 60 lesions in 9 enrolled patients with lung cancer [36]. Another study assessed the effect of segmentation on MTV repeatability using various SUV thresholds and found the best performing segmentation threshold had an ICC of 0.99 and RC of 36.1% [37].

The choice of cooling method, which has been the most common method of activating BAT, potentially may influence FDG repeatability in brown fat studies. The influence of cooling method choice on active BAT repeatability has not been studied, but it has been theorized that “individualizing” (i.e. each individual is cooled to a different extent in order to reach the just-above-shivering threshold) the cooling method will cause maximal non-shivering thermogenesis and, therefore more consistent FDG uptake in BAT [38, 39]. Although the ice-block technique used here can be considered individualized, it potentially may not be as optimized in terms of modulating body temperature as are other methods. Many studies of human BAT have accomplished activation by means of only a cooled room or a cooled room with a fan, which are considered “fixed” methods [20, 40]. More commonly used individualized methods utilize a cooling blanket/suit/vest or a climate tent [41, 42]. The advantage of these methods is precise and real-time temperature control, so that once shivering starts, body temperature can be quickly raised above the non-shivering threshold. The ice-block method used here can certainly cool a region of the body and activate BAT, but once the shivering point has been reached, the only remedy is reducing the exposure to the cold ice-block, as opposed to more sensitive methods that can also introduce warmth [43]. This inability to raise body temperature may result in prolonged shivering, which could impact FDG uptake in BAT, especially if the shivering occurs after administration of FDG. Future studies may focus on the impact of cooling method choice on the repeatability of FDG uptake in activated BAT. In our cases, we did not see intense FDG uptake in muscle suggesting shivering was not a major cause of an altered FDG biodistribution.

Several other factors may have contributed to the relatively low repeatability found in our study, compared to FDG repeatability seen in the oncologic setting. The method used to detect shivering in this study was by researcher observation or self-report by the volunteer. A superior method, which was beyond the scope of this study, could be to use electromyography (EMG), which can potentially detect shivering earlier than observation or report. Another limitation of this study is that menstrual cycle information for these participants is not known. Potentially, menstrual cycle phase may impact BAT activity and such information should be collected in future studies. Given the lower reported variability of FDG PET measures of tumors on test and re-test, it is probable that much of the greater variance is not due to scanner factors, but rather due to the dynamic metabolic characteristics of the brown adipose tissue using our activation method, thus contributing to the variability in FDG uptake between studies. Although we attempted to image volunteers twice under precisely the same conditions, it is practically difficult to ensure variables such as patient preparation (e.g. fasting duration and types of food eaten prior to the study) and exercise levels are totally constant between test and retest imaging studies. Though each volunteer was imaged during the same season, it is possible that daily variations in outdoor temperature could have impacted BAT activity during test and retest sessions [44]. Larger sample sizes may also be necessary to more effectively determine FDG repeatability in brown fat. It is also possible that a more sophisticated cooling approach will result in lower variability than we observed. We are assessing a cooling blanket approach as an alternative to the simple ice block/cold room method used here.

## Conclusion

FDG PET/CT can commonly detect active BAT under conditions of controlled cold, ice-block, intervention in humans. While the qualitative presence/absence of BAT appears reasonably correlated between test and retest imaging, quantitative assessments of SUV and BMV vary substantially on test-retest using our regional cold BAT activation approach. The considerable variation in estimates of BAT with FDG PET with our ice-block method implies sample sizes will need to be large in studies quantifying physiological changes in BAT volume and metabolic activity related to treatment and support the highly dynamic metabolic character of this tissue. It is possible that more sophisticated cooling methods will have more reproducible results.
